# Oxygen Depletion and the Role of Cellular Antioxidants in FLASH Radiotherapy: Mechanistic Insights from Monte Carlo Radiation-Chemical Modeling

**DOI:** 10.3390/antiox14040406

**Published:** 2025-03-28

**Authors:** Israth Rabeya, Jintana Meesungnoen, Jean-Paul Jay-Gerin

**Affiliations:** Department of Medical Imaging and Radiation Sciences, Faculty of Medicine and Health Sciences, Université de Sherbrooke, 3001, 12th Avenue Nord, Sherbrooke, QC J1H 5N4, Canada; israth.rabeya@usherbrooke.ca (I.R.); jintana.meesungnoen@usherbrooke.ca (J.M.)

**Keywords:** FLASH radiotherapy (FLASH-RT), cell water radiolysis, high dose rate, peroxyl radicals, glutathione, ascorbate, nitric oxide, α-tocopherol, Monte Carlo multi-track chemical modeling, radiation chemical yields (*G* values), radiolytic oxygen depletion (ROD) hypothesis

## Abstract

FLASH radiotherapy is a novel irradiation modality that employs ultra-high mean dose rates exceeding 40–150 Gy/s, far surpassing the typical ~0.03 Gy/s used in conventional radiotherapy. This advanced technology delivers high doses of radiation within milliseconds, effectively targeting tumors while minimizing damage to the surrounding healthy tissues. However, the precise mechanism that differentiates responses between tumor and normal tissues is not yet understood. This study primarily examines the ROD hypothesis, which posits that oxygen undergoes transient radiolytic depletion following a radiation pulse. We developed a computational model to investigate the effects of dose rate on radiolysis in an aqueous environment that mimics a confined cellular space subjected to instantaneous pulses of energetic protons. This study employed the multi-track chemistry Monte Carlo simulation code, IONLYS-IRT, which has been optimized to model this radiolysis in a homogeneous and aerated medium. This medium is composed primarily of water, alongside carbon-based biological molecules (RH), radiation-induced bio-radicals (R^●^), glutathione (GSH), ascorbate (AH^−^), nitric oxide (^●^NO), and α-tocopherol (TOH). Our model closely monitors the temporal variations in these components, specifically focusing on oxygen consumption, from the initial picoseconds to one second after exposure. Simulations reveal that cellular oxygen is transiently depleted primarily through its reaction with R^●^ radicals, consistent with prior research, but also with glutathione disulfide radical anions (GSSG^●−^) in roughly equal proportions. Notably, we show that, contrary to some reports, the peroxyl radicals (ROO^●^) formed are not neutralized by recombination reactions. Instead, these radicals are rapidly neutralized by antioxidants present in irradiated cells, with AH^−^ and ^●^NO proving to be the most effective in preventing the propagation of harmful peroxidation chain reactions. Moreover, our model identifies a critical dose rate threshold below which the FLASH effect, as predicted by the ROD hypothesis, cannot fully manifest. By comparing our findings with existing experimental data, we determine that the ROD hypothesis alone cannot entirely explain the observed FLASH effect. Our findings indicate that antioxidants might significantly contribute to the FLASH effect by mitigating radiation-induced cellular damage and, in turn, enhancing cellular radioprotection. Additionally, our model lends support to the hypothesis that transient oxygen depletion may partially contribute to the FLASH effect observed in radiotherapy. However, our findings indicate that this mechanism alone is insufficient to fully explain the phenomenon, suggesting the involvement of additional mechanisms or factors and warranting further investigation.

## 1. Introduction

### 1.1. On the FLASH Effect

Radiation therapy (RT) is a fundamental component of modern cancer treatment, employed in conjunction with surgery, chemotherapy, and immunotherapy. Utilizing ionizing radiation, RT targets and eradicates cancer cells, constituting a vital part of the standard oncological care protocol [1]. Since the introduction of X-rays, RT has seen remarkable technological progress. Innovations such as intensity-modulated RT and stereotactic body RT have significantly improved the precision of radiation delivery to tumors, effectively sparing surrounding healthy tissues from damage. A key challenge in RT is to administer cytotoxic doses of radiation that are potent enough to treat cancer effectively while minimizing the risk of severe acute and chronic side effects in nearby normal tissue. In response to this challenge, clinical radiotherapy has recently shifted its focus towards ultra-high dose rate irradiation, known as FLASH irradiation [2,3,4], which delivers doses at mean rates exceeding ~40 to 150 gray per second (Gy/s). This innovative approach has been found to markedly reduce adverse effects on healthy tissues while maintaining the anti-tumor efficacy seen with standard (STD) clinical dose rates, which are typically ~0.5 to 5 Gy per minute (i.e., several thousand times slower than FLASH irradiation; see, e.g., [5,6,7,8,9,10,11,12,13,14,15,16,17,18,19,20,21]).

The evidence that FLASH-RT can spare normal tissues, thus challenging long-established principles of classical radiobiology, is supported by multiple preclinical studies [22,23,24]. These investigations, conducted both in vitro and in vivo, have involved cell cultures and animal models, primarily employing electron linear accelerators. More recently, the protective benefits of FLASH-RT have been demonstrated using megavoltage photons [25,26,27,28], cyclotron-based protons [29,30,31,32,33], helium [34], and carbon ions [35,36,37], significantly broadening its potential applications.

A landmark turning point in the use of FLASH-RT occurred with the treatment of the first human patient, who had cutaneous lymphoma, using a 5.6 MeV electron FLASH beam. This treatment delivered an instantaneous dose rate of 10^6^–10^7^ Gy/s and resulted in the complete eradication of the tumor mass with minimal toxicity to the surrounding healthy tissue, highlighting the therapeutic potential of FLASH-RT [38,39]. Subsequently, a pioneering first-in-human trial explored the use of FLASH proton therapy for managing painful bone metastases [33]. The outcomes indicated that this novel treatment provided pain relief comparable to STD-RT without inducing any unexpected adverse effects, further confirming the clinical promise of FLASH-RT to enhance cancer treatment protocols while reducing side effects. Although there is considerable excitement surrounding these advancements, the precise molecular mechanisms underlying the sparing of normal tissue and the differential responses of tumor and normal tissues to FLASH-RT remain largely undefined. Understanding these aspects is essential for clinically translating the FLASH effect, and it is the primary focus of this study.

Several hypotheses have been proposed to explain the mechanisms underlying the FLASH effect. Among the most notable are the oxygen depletion hypothesis, the reactive oxygen species (ROS) and free radical interaction hypothesis, the DNA integrity hypothesis, the mitochondrial hypothesis, and the immune regulation hypothesis. An expanding body of research investigates these mechanisms, with recent review articles offering comprehensive evaluations [13,14,17,21,24]. Notably, these mechanisms may be interconnected, reflecting the intricate cellular and molecular biology that underlies the FLASH effect [21].

The oxygen depletion hypothesis, also known as the ROD hypothesis [5,40,41,42,43,44,45,46,47], is widely regarded as the most prominent, proposing that the FLASH effect arises, at least in part, from the transient radiolytic consumption or “depletion” of intracellular oxygen. First proposed nearly half a century ago based on experiments with cells irradiated at ultra-high dose rates [48,49,50,51], the ROD hypothesis posits that oxygen consumed during a brief period of radiation exposure cannot be replenished quickly enough through diffusion from blood vessels to individual cells. This leads to a temporary state of acute hypoxia, which in turn temporarily enhances the cells’ resistance to radiation. This phenomenon, characterized by decreased radiosensitivity, occurs selectively in oxygenated normal cells, but is absent in tumor cells, which already exist in a poorly oxygenated environment. Despite its widespread acceptance, the ROD hypothesis continues to be debated [52,53,54,55], specifically concerning whether the amount of O_2_ depleted during pulse irradiation is enough to induce significant hypoxia in initially well-oxygenated tissues in vivo, thereby fully accounting for the protective effects observed with FLASH-RT.

Our work is mainly focused on the first two hypotheses which are based mainly on chemical reactions between primary products formed during water radiolysis and either oxygen and/or biological molecules and endogenous antioxidants.

### 1.2. Water Radiolysis in Cellular Environments

Water is the primary component in living cells and tissues by weight, making an understanding of water radiolysis essential for comprehending radiobiological effects. Quantitatively, the main products of the radiolysis of pure deaerated water include hydrated electrons (e^−^_aq_), hydrogen atoms (H^●^), molecular hydrogen (H_2_), hydroxyl radicals (^●^OH), hydrogen peroxide (H_2_O_2_), hydronium ions (H_3_O^+^), and hydroxide ions (OH^−^) (see, e.g., [56,57,58,59]). These products initially form within a nonhomogeneous track structure where ^●^OH and e^−^_aq_ radicals are most abundant. Under aerated conditions, e^−^_aq_ and H^●^ atoms quickly (within microseconds) convert to superoxide/hydroperoxyl radicals (O_2_^●−^/HO_2_^●^), with O_2_^●−^ in pH-dependent equilibrium with its conjugate acid (p*K*_a_ ≈ 4.8 at 25 °C) [60]. As a result, at physiological pH, the main reactive species when homogeneity is achieved are O_2_^●−^, ^●^OH, and H_2_O_2_. H_2_ plays only a minor role in the radiolysis of aqueous solutions, with most of it escaping from the solution.

In cellular environments, the situation is markedly different. Firstly, while reactions involving e^−^_aq_ and H^●^ with O_2_ are prominent in pure, aerated water radiolysis, they are much less frequent in cells. This reduction is largely due to the high concentration of intracellular bio-organic molecules (collectively referred to as RH), such as DNA, proteins, and lipids [47,61,62,63], which effectively compete with O_2_ for the capture of these species [40,47,62,63,64,65,66,67]. For instance, the scavenging power for the reaction of e^−^_aq_ with these biomolecules is estimated to be 3.4 × 10^8^ s^−1^ [61], nearly 1000 times greater than that for 30 μM oxygen—a typical concentration in most normal tissues [56]—estimated at 5.7 × 10^5^ s^−1^. Here, “scavenging power” refers to the product of the scavenger’s concentration and its reaction rate constant with e^−^_aq_. This notable difference in scavenging power between cellular components and oxygen within cells was underscored by Wardman [64] and Favaudon et al. [47], and further substantiated through Monte Carlo multi-track chemistry simulations by Sultana et al. [68]. This challenges the previous model of treating a cell merely as a “bag” of pure, aerated water (see, e.g., [69,70,71,72,73]). Indeed, during FLASH irradiation, the temporary depletion of O_2_ by e^−^_aq_/H^●^ is very low in a cellular or tissue environment.

Secondly, in irradiated biological systems, carbon-centered organic radicals (represented by R^●^) are generated through two main pathways: the deprotonation of intermediate radical cations (RH^●+^) that initially arise from the direct ionization of biomolecules or via hydrogen abstraction reactions involving RH. The latter pathway commonly involves ^●^OH radicals produced through the radiolysis of water [56,57,74]:

RH + ^●^OH → R^●^ + H_2_O. (1)

The rate constant for this reaction typically ranges from 10^8^ to 10^9^ M^−1^ s^−1^, influenced by the specific structure of RH and the type of hydrogen atoms it contains. Under oxidative stress, if cellular antioxidant defenses like glutathione, vitamin C (ascorbate), or vitamin E (α-tocopherol)—which donate hydrogen atoms to repair molecular damage—are inadequate, R^●^ radicals can react with O_2_ to produce peroxyl radicals (ROO^●^). These reactions occur at rates approaching the diffusion-controlled limit [75,76,77,78]:

R^●^ + O_2_ → ROO^●^, *k* ~ 2 × 10^9^ M^−1^ s^−1^. (2)

Peroxyl radicals are more potent oxidizers than their precursor radicals. As chain-propagating species, they particularly target polyunsaturated lipids from which they abstract H^●^ atoms, leading to hydroperoxide (ROOH) formation—a key step in lipid peroxidation processes [75,79,80,81,82]. These ROO^●^ radicals can also form in various cellular macromolecules, including proteins and DNA (see, e.g., [83]). Once generated, peroxyl radicals permanently alter the original molecules, a process known as the “fixation” of damage by oxygen [84,85]. This irreversible damage presents formidable obstacles for cellular repair mechanisms, often making the restoration of damaged structures challenging or unachievable.

In other words, the physiological levels of oxygen and antioxidants are pivotal in shaping the outcomes for intermediate radicals in biomolecules. Labarbe et al. [47,62] emphasized that any cellular processes which either reduce the radiolytic production of ROO^●^ radicals or shorten their lifetime could protect healthy tissues from the detrimental effects of radiation. This insight prompted these authors [47] and others [64,65,66] to suggest that radical–radical combination and recombination (ROO^●^-ROO^●^, ROO^●^-R^●^, or R^●^-R^●^) could account for the protective effects seen with FLASH irradiation, a hypothesis supported by the high dose rates characteristic of FLASH-RT. From a radiation–chemical perspective, high dose rate irradiation increases transient radical concentrations through enhanced multi-track chemistry [57,86,87], potentially raising the proportion of peroxyl radical recombination during FLASH irradiation relative to STD-RT. However, contrary to these expectations, the model presented here indicates that such recombination reactions have minimal impact on outcomes within the first second after irradiation. Comparable findings were also reported by Baikalov et al. [88]. This limited effect is partly due to the relatively slow nature of these reactions, with rate constants ranging from ~10^4^ M^−1^ s^−1^ for (ROO^●^ + ROO^●^) to ~10^8^ M^−1^ s^−1^ for (R^●^ + R^●^).

### 1.3. Investigating the Role of Cellular Antioxidants in FLASH-RT

Building on these findings and Wardman’s discussions [64], we propose that cellular antioxidants, rather than ROO^●^ and R^●^ radical–radical recombination reactions, are central to mediating the FLASH radiotherapeutic response. The antioxidants considered in this study include nitric oxide (^●^NO, also known as nitrogen monoxide), glutathione (GSH), ascorbate (AH^−^), and α-tocopherol (TOH). Below, we explore the significance and relevance of each of these antioxidants in addressing the problem at hand.

(1) ^●^NO, a naturally occurring free radical (note that the unpaired electron is located on the nitrogen atom) produced endogenously in most mammalian cells, is synthesized by a family of enzymes known as nitric oxide synthases (NOS), which are divided into constitutive (cNOS) and inducible (iNOS) isoforms. Particularly pertinent to our research, iNOS can be activated by inflammatory stimuli like physiological stress, often triggered by events that generate excessive reactive oxygen/nitrogen species (ROS/RNS) such as those occurring during high dose rate (FLASH) irradiation. This leads to the production of ^●^NO in supraphysiological concentrations, potentially reaching micromolar levels or sustained over extended periods of time (see, e.g., [82,89,90,91,92,93,94] and cited references). This increased ^●^NO production can be viewed as a protective or adaptive response, where ^●^NO acts as an antioxidant by scavenging free radicals produced by radiation, thereby reducing oxidative stress and potentially minimizing DNA damage.

However, in the presence of superoxide radicals (O_2_^●−^), ^●^NO can also contribute to cytotoxic effects by generating highly reactive peroxynitrite (ONOO^−^, p*K*_a_ = 6.8 at 37 °C) [92,95,96,97]:

^●^NO + O_2_^●−^ → ONOO^−^, *k* = 1.9 × 10^10^ M^−1^ s^−1^. (3)

Moreover, nitric oxide is involved in one of the fastest known reactions with organic peroxyl radicals, resulting in the formation of organic peroxynitrites (or pernitrites) [76,82,98,99,100]:

ROO ^●^ + ^●^NO → ROONO, *k* ~ 2 × 10^9^ M^−1^ s^−1^. (4)

Organic peroxynitrites are generally unstable and likely non-reactive. In fact, ROONO may undergo the homolytic cleavage of the O–O bond, resulting in a caged radical pair that can either recombine to form a more stable organic nitrate, RONO_2_, or dissociate into free alkoxyl (RO^●^) and nitrogen dioxide (^●^NO_2_) radicals. This dissociation pathway could theoretically increase lipid peroxidation, a process that contradicts numerous studies underscoring ^●^NO’s antioxidant properties [99,100,101]. However, it has been suggested that RO^●^ could react with ^●^NO to give RONO, thus inhibiting further oxidative reactions. Moreover, ^●^NO_2_ can react with ^●^NO to form dinitrogen trioxide, which subsequently hydrolyzes to nitrite anion [99].

Due to its high lipophilicity, charge neutrality, and small molecular size, ^●^NO easily diffuses through cell membranes and accumulates in lipophilic compartments. It acts as an effective lipid-soluble, chain-breaking antioxidant, not only inhibiting lipid peroxidation propagation but also preserving lipophilic antioxidants, such as α-tocopherol [102], thereby enhancing the overall cellular resistance to radiation. Notably, in FLASH-irradiated cells, reaction (4) predominates over reaction (3) because O_2_ is more likely to be consumed in forming ROO^●^ rather than reacting with e^−^_aq_ or H^●^ to form O_2_^●−^, due to the high concentrations of competing intracellular scavengers for e^−^_aq_/H^●^, as previously discussed [64,68].

These rapid reactions between peroxyl radicals and ^●^NO are critical in regulating lipid oxidation processes in cell membranes, lipoproteins, and other lipid-containing structures, which are central to this study. These reactions are particularly significant for their potential contribution to the FLASH effect, where ^●^NO’s antioxidant capabilities could play a crucial role in synergy with other key cellular antioxidants like glutathione, ascorbate, and α-tocopherol.

In this study, a nitric oxide concentration of [^●^NO] = 1 μM was used. As previously mentioned, this concentration exceeds the estimated steady-state ^●^NO levels in normal tissue, which typically range from ~0.02 to 0.1 μM, reflecting the specific conditions associated with the high dose rate effects of FLASH irradiation [82,89,90,91,92,93,94,103,104].

(2) Thiols are well-known for their role in protecting cells from damage caused by ROS/RNS [75,82]. In this study, we selected glutathione (GSH) as the representative thiol antioxidant because of its high prevalence in mammalian tissues, where it is commonly found at millimolar concentrations (1–10 mM) [105]. Although GSH is primarily synthesized in the cytoplasm, it is also abundant in the cell nucleus. Notably, GSH exhibits moderate reactivity with ROO^●^ peroxyl radicals, leading to the formation of the glutathione thiyl radical GS^●^ [77,106]:

ROO^●^ + GSH → GS^●^ + ROOH, *k* = 5 × 10^4^ M^−1^ s^−1^ (5)

This radical can further react with O_2_, ^●^NO, and ascorbate (AH^−^) or dimerize to produce the oxidized form of glutathione, glutathione disulfide (GSSG) (see, e.g., [82]). To simulate the intracellular environment in our cellular model, we used a concentration of [GSH] = 6.5 mM. This concentration aligns with the values reported in the existing literature [62,65,67,105,107,108], highlighting GSH’s critical role in mitigating oxidative stress and maintaining the cellular redox balance.

(3) Ascorbic acid (vitamin C, AH_2_), along with glutathione, is a potent water-soluble biological antioxidant and free-radical scavenger. It is typically present in most cells at relatively high concentrations, ranging from 1 to 2.5 mM [109,110]. Ascorbic acid readily loses a proton to form the ascorbate anion [111,112]:

AH_2_ → AH^−^ + H^+^ (p*K*_a,1_ = 4.04; p*K*_a,2_ = 11.3), (6)

making the protonated form negligible at physiological pH. Ascorbate anions react with ROO^●^ according to [63,76,77,109]:

ROO^●^ + AH^−^ → A^●−^ + ROOH, *k* = 2.2 × 10^6^ M^−1^ s^−1^. (7)

Ascorbate radicals (A^●−^), also known as ascorbyl radicals, generated in reaction (7), are relatively unreactive. They primarily undergo disproportionation among themselves, serving as effective free radical chain terminators [113,114,115]. Importantly, A^●−^ does not readily engage in addition reactions with O_2_ (*k* < 5 × 10^2^ M^−1^ s^−1^), thus preventing the formation of harmful peroxyl radicals [115,116]. Similarly, these radicals exhibit minimal reactivity with ^●^NO [110]. In our simulations, we employed an ascorbate concentration of [AH^−^] = 1 mM.

(4) Vitamin E is recognized as the first and most potent lipid-soluble chain-breaking antioxidant [117]. α-tocopherol (TOH) is known to react with peroxyl radicals within lipid-rich environments, such as membranes or lipoproteins, effectively preventing lipid peroxidation. Remarkably, the tocopheroxyl radicals (TO^●^) produced in this reaction can be regenerated into functional vitamin E by ascorbate (AH^−^), thereby providing sustained antioxidant protection [82,101,114,118,119]. Furthermore, note that there is no direct reaction between nitric oxide and TO^●^ radicals [101]. For our simulations, we utilized an α-tocopherol concentration of [TOH] = 0.2 mM [65].

## 2. Materials and Methods

### 2.1. Determining the Effects of Dose Rates Using the ‘Instantaneous Pulse’ (Dirac) Model

In this study, we employed our newly developed multi-track irradiation model to explore the impact of high dose rates on water radiolysis using single, instantaneous pulses of *N* incident 300-MeV protons [68,69,87,120]. These protons mimic the low linear energy transfer (LET) characteristic of ^60^Co γ-ray Compton electrons or fast (e.g., MeV) electrons, with an LET of ~0.3 keV/μm [121]. Notably, in the absence of dose rate effects (i.e., no interaction between tracks), their track structure initially comprises small, well-separated Magee-type “spurs”—roughly spherical clusters of radiolytic species distributed along the radiation path [57,58,59]).

Briefly, these monoenergetic protons simultaneously strike the water surface perpendicularly, covering a circular area with radius *R*_o_, as illustrated in Figure 1 of Alanazi et al. [69]. Known as the ‘instantaneous pulse’ or Dirac model, this method assumes a pulse duration of zero [122], leading to the immediate formation of all chemical species.

Using energetic protons offers the advantage of their essentially rectilinear trajectories, allowing the definition of a cylindrical beam geometry upon entry. Within this configuration, all proton tracks are aligned with the cylinder’s axis for the entire chosen track length. This setup closely mirrors the conditions encountered in our long-standing Monte Carlo simulations of water radiolysis at low dose rates (see, e.g., [59] for a review), but instead of simulating a single proton track, we simultaneously simulate *N* interactive tracks. In this particular setup, the incident proton “fluence” (number of impacting protons per unit area) is calculated as *N*/π*R*_o_^2^.

By varying *N*, the number of protons per pulse, we directly investigate how different dose rates affect our system. This study examines four values of *N*: 20, 30, 40, and 50. Based on our prior calibration of *N* in relation to dose rate (refer to Figure 3B in Alanazi et al. [69]), *N* = 20 corresponds to an absorbed dose rate of ~10^6^–10^7^ Gy/s under our irradiation conditions.

We define “time zero” as the moment the *N* incident protons reach the front of the cylinder.

### 2.2. Monte Carlo Multi-Track Chemistry Simulations Using the IONLYS-IRT Code

In the context of water radiolysis, our IONLYS program [59,123] simulates the initial physical and physicochemical stages of radiation action within a 3D environment, capturing events up to ~1 picosecond (ps) in track development. It accurately models each event, detailing all fundamental physical interactions associated with energy deposition and the transformation of local physical products into various initial radical and molecular radiolysis products. These products include e^−^_aq_, H^●^, H_2_, ^●^OH, H_2_O_2_, H^+^, and OH^−^, among others [59]. This program generates a detailed and highly nonhomogeneous spatial distribution of reactants, setting the stage for the subsequent chemical stage of radiolysis that begins after ~1 ps. In this third stage, the various radiolytic products diffuse randomly from their origin points, governed by their diffusion coefficients. They react either with each other or, competitively, with uniformly distributed solutes in the system. Our IRT program [124] handles this stage using the “independent reaction times” (IRT) method [125,126,127], a stochastic simulation technique that efficiently calculates reaction times without needing to track individual diffusion trajectories. The accuracy of this program in delivering chemical yields (or *G* values) has been validated under various irradiation conditions through comparisons with detailed “step-by-step” Monte Carlo simulations, which closely track the trajectories of diffusing reactive species [128]. Additionally, the IRT program is particularly effective in modeling reactions over extended periods of time when tracks have dissipated and the radiolytic products are uniformly distributed within the solution.

Throughout this article, *G* values are expressed as the number of molecules formed or consumed per 100 eV of absorbed radiation energy. The conversion of these values to SI units is as follows: 1 molecule per 100 eV ≈ 0.103364 μmol/J [57].

### 2.3. Our Irradiated Cell Water Model: Proposed Chemical Reaction Scheme

In our approach, we model a cell as a homogeneous aqueous medium, thereby ignoring cellular heterogeneity. Cells are inherently complex, consisting of various compartments and structures like the cytoplasm, nucleus, mitochondria, and phospholipid membranes, each characterized by unique chemical and physical properties. As pointed out by Wardman [63,64,129], while homogeneous kinetics provide a practical modeling framework, this approach may overlook important aspects of cellular behavior and interactions. Despite these limitations, we argue that our basic cell water model, which utilizes homogeneous kinetics, is instrumental for identifying primary reactants and key reactions in cells exposed to high dose rates, thus advancing our understanding of the chemical mechanisms driving the FLASH effect.

The model assumes cellular composition based on the following:

- Dissolved oxygen, typically around 30 μM in well-oxygenated mammalian tissues, as reported in [56,68,103].
- Carbon-based biomolecules (RH), such as DNA and RNA (present in much lower concentrations than proteins and lipids), proteins (the primary components by weight in most cells), free amino acids, free nucleotides, and lipids (mainly in cell membranes), typically occur at a concentration of [RH] ~ 1 M [62,130]. This concentration has been previously utilized in the literature (e.g., see [62,67]). In our simulations, 1 M denotes the ‘oxidizable’ substrate concentration in a living system, as defined by Qian and Buettner [130]. Nevertheless, a 1 M concentration of bio-organic molecules may appear high when averaged across an entire cell. To account for some cellular heterogeneity, we also tested a 0.5 M concentration, both for comparison and to evaluate the sensitivity of the results to [RH] variations.
- For simplicity, as noted above, our model does not distinguish between these macromolecular constituents. We acknowledge that using a single RH species poorly captures variations in radiation-induced cellular damage, particularly the differences between DNA and lipids. To improve accuracy, we are currently developing a more detailed compartmental cell model akin to those proposed by Hu et al. [65] and Babbs and Steiner [106].
- Under the specific conditions of FLASH irradiation employed in this study—a ~30 Gy dose delivered with 300-MeV protons (LET ~ 0.3 keV/μm) at an instantaneous dose rate of ~10^6^–10^7^ Gy/s [68,69]—we estimate an initial bio-radical (R^●^) concentration of ~2.5 μM. These bio-radicals originate from the ‘direct’ ionization of RH, followed by the deprotonation of the resulting radical cations (RH^●+^). This concentration estimate is derived from direct action yields reported in previous studies [40,62,65].

We supplemented the reaction scheme in our IONLYS-IRT track chemistry computer code, which models the radiolysis of pure, deaerated liquid water at 25 °C under 300-MeV proton irradiation (refer to Table 14.1 in [59])—a condition that mimics cobalt-60 γ-ray exposure (as previously discussed) to incorporate 48 reactions relevant to the irradiated cell water model being studied. These specific reactions are listed in Table 1.

**Table 1:** Reaction scheme and rate constants (*k*, in M^−1^ s^−1^) for simulating radiolysis of our cell water model at room temperature *^a,b^*.

Symbol	Reaction	*k*	References
(R1)	RH + e^−^_aq_ → (RH)^•−^	1.5 × 10^9^	[61,131,132]
(R2)	RH + H^•^ → RH(+H)^•^	8 × 10^7^	[61,131,132,133]
(R3)	O_2_ + e^−^_aq_ → O_2_^•−^	1.9 × 10^10^	[74]
(R4)	O_2_ + H^•^ → HO_2_^•^ → O_2_^•−^ + H^+^ (p*K*_a_ = 4.8) *^c^*	2.1 × 10^10^	[60,74]
(R5)	O_2_^•−^ + O_2_^•−^ + 2H^+^ → O_2_ + H_2_O_2_ *^d^*	4 × 10^9^	[82,92,134]
(R6)	H_2_O_2_ + H_2_O_2_ → 2H_2_O + O_2_ *^e^*	2 × 10^7^	[82]
(1)	RH + ^•^OH → R^•^ + H_2_O	5 × 10^8^	[56,57,61,74,132]
(2)	R^•^ + O_2_ → ROO^•^	2 × 10^9^	[75,76,77,78]
(R7)	R^•^ + R^•^ → R–R	10^8^	[62,106]
(R8)	R^•^ + ROO^•^ → ROOR	5 × 10^7^	[106]
(R9)	ROO^•^ + ROO^•^ → products	10^5^	[62,65,75]
(R10)	ROO^•^ + RH → ROOH + R^•^	1.3 × 10^3^	[62,65,75]
(R11)	ROO^•^ + O_2_^•−^ + H^+^ → ROOH + O_2_	5 × 10^7^	[82,106,135]
(R12)	e^−^_aq_ + ROOH → RO^•^ + OH^−^	10^10^	[56]
(R13)	RO^•^ + RH → ROH + R^•^	5 × 10^4 *f*^	[57]
(5)	ROO^•^ + GSH → GS^•^ + ROOH *^g^*	5 × 10^4^	[77,106]
(R14)	R^•^ + GSH → GS^•^ + RH	5.6 × 10^6^	[65]
(R15)	GSH + e^−^_aq_ + H^+^ → G^•^ + H_2_S	4.5 × 10^9^	[74]
(R16)	GSH + H^•^ → GS^•^ + H_2_ *^h^*	1.8 × 10^9^	[136]
(R17)	GSH + ^•^OH → GS^•^ + H_2_O	1.4 × 10^10^	[74,106,137,138]
(R18)	GS^•^ + O_2_ → GSOO^•^	2 × 10^9^	[65,139,140,141]
(R19)	GSOO^•^ → GS^•^ + O_2_	6.2 × 10^5^	[65,108,130]
(R20)	GSOO^•^ + GSH → GSO^•^ + GSOH	2 × 10^6^	[108]
(R21)	GS^•^ + GS^•^ → GSSG *^i^*	7.5 × 10^8^	[82,139,142]
(R22)	GS^•^ + GSH → GSSG^•−^ + H^+^	3.5 × 10^8^	[65,136,137,142]
(R23)	GSSG^•−^ + O_2_ → GSSG + O_2_^•−^	5.1 × 10^8^	[82,139,140,141]
(R24)	GSSG + e^−^_aq_ → GSSG^•−^	3.7 × 10^9^	[74]
(R25)	GSSG + H^•^ → GSH + GS^•^	10^10^	[74]
(R26)	GSSG + ^•^OH → GSSG^•+^ + OH^−^	1.7 × 10^10^	[136]
(R27)	GSSG^•+^ + GSSG^•+^ → GSSG^2+^ + GSSG	2.5 × 10^9^	[136]
(R28)	GS^•^ + ^•^NO → GSNO	3 × 10^9^	[142]
(R29)	GS^•^ + GSNO → GSSG + ^•^NO	1.7 × 10^9^	[142]
(R30)	GS^•^ + AH^−^ → GSH + A^•− *j,k*^	6 × 10^8^	[65,82,139,140]
(R31)	R^•^ + AH^−^ → RH + A^•−^	10^7^	[65]
(7)	ROO^•^ + AH^−^ → ROOH + A^•−^	2.2 × 10^6^	[63,76,77,109,113,114,115]
(R32)	RO^•^ + AH^−^ → ROH + A^•−^	1.6 × 10^9^	[114,115]
(R33)	AH^−^ + O_2_^•−^ + H^+^ → A^•−^ + H_2_O_2_	2.7 × 10^5^	[82,114,115,116,139]
(R34)	AH^−^ + ^•^OH → A^•−^ + H_2_O	1.1 × 10^10^	[74,82,114,115]
(R35)	A^•−^ + A^•−^ + H^+^ → AH^−^ + A *^l^*	2.8 × 10^5^	[113,114,115]
(R36)	A^•−^ + O_2_^•−^ + 2H^+^ → A + H_2_O_2_	2.6 × 10^8^	[113,114]
(R37)	^•^OH + TOH → TO^•^ + H_2_O *^m^*	10^10^	[106]
(R38)	R^•^ + TOH → RH + TO^•^	2.5 × 10^6^	[65,106]
(R39)	ROO^•^ + TOH → ROOH + TO^•^	5 × 10^5^	[56,65,82,99,101,106]
(R40)	TO^•^ + AH^−^ → TOH + A^•−^	1.3 × 10^7^	[114]
(3)	NO^•^ + O_2_^•−^ → ONOO^− *n*^	1.9 × 10^10^	[95,96,97,98,143,144,145]
(R41)	NO^•^ + ^•^OH → HNO_2_	10^10^	[82]
(R42)	R^•^ + ^•^NO → RNO	2 × 10^9^	[129]
(4)	ROO^•^ + ^•^NO → ROONO	2 × 10^9 *o*^	[98,99,100,101,102]

*^a^* The rate constants quoted here for reactions between ions are based on conditions of infinite dilution, where ion–ion interactions are absent. In the IRT program, we disregarded the effects of ionic strength on all these reactions. *^b^* For the model presented here, much of the chemistry is well established. However, there are uncertainties associated with the reaction rate constants. These uncertainties can vary significantly, ranging from a few percent to a factor of ten or more in some instances. *^c^* Favored at physiological pH. *^d^* Enzymatic dismutation of O_2_^•−^ catalyzed by Cu/Zn superoxide dismutase (SOD). *^e^* In the presence of catalase. *^f^* Estimated from [57]. *^g^* GS^•^: Glutathione thiyl radical. *^h^* Assuming that the rate constant for this reaction is similar to the rate at which H^•^ atoms react with cysteine (CysSH) in aqueous solution (*k* ~ 1–4 × 10^9^ M^−1^ s^−1^) (refer to Table 2 in [136]). *^i^* GSSG: Glutathione disulfide, also commonly referred to as oxidized glutathione. *^j^* AH^−^: Monohydrogen ascorbate anion. *^k^* A^•−^: Ascorbate or ascorbyl radicals. *^l^* Dehydroascorbic acid (A) produced by the disproportionation of two ascorbate radicals. *^m^* TO^•^: Tocopheroxyl radical. *^n^* ONOO^−^: peroxynitrite. *^o^* Nitric oxide is 10^4^–10^5^ times more effective as a peroxyl radical scavenger than α-tocopherol [99].

## 3. Results and Discussion

**Figure 1:**
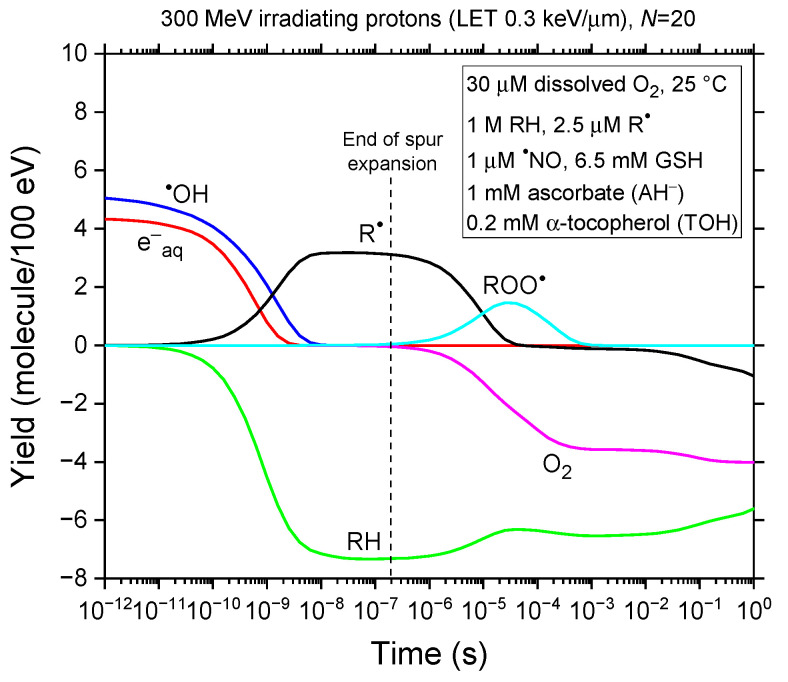
Time evolution of the yields of key reactive species (e^−^_aq_, ^●^OH, RH, R^●^, ROO^●^, and O_2_) from our IONLYS-IRT Monte Carlo track chemistry simulations of radiolysis in our modeled cell-like water, which contains 30 μM dissolved oxygen at 25 °C, covering a time span from 1 ps to 1 s. Simulations were conducted under representative FLASH irradiation conditions using 300-MeV incident protons (LET ~ 0.3 keV/μm), delivering ~30 Gy at an instantaneous dose rate of ~10^6^–10^7^ Gy/s, corresponding to *N* = 20 irradiating protons per pulse. For reference, the dashed line at ~0.2 μs indicates the transition from nonhomogeneous spur kinetics to homogeneous kinetics in the bulk solution in the absence of dose rate effects [57,146].

**Figure 2:**
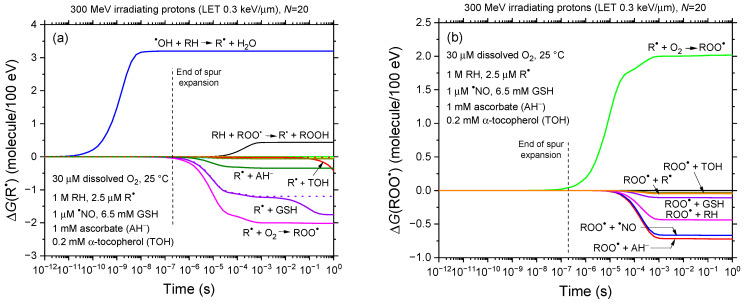
Time-dependent extents Δ*G*(R^●^) (panel **a**) and Δ*G*(ROO^●^) (panel **b**), expressed in molecules per 100 eV, for various reactions contributing to *G*(R^●^) and *G*(ROO^●^) (shown in Figure 1) from 1 ps to 1 s, based on our Monte Carlo simulations. The irradiation conditions are the same as those described in the caption of Figure 1. Panel (**a**) shows that R^●^ primarily forms at early times through reaction (1) (RH + ^●^OH → R^●^ + H_2_O) and, much later and to a significantly lesser extent, through reaction (R10) (RH + ROO^●^ → R^●^ + ROOH). Its consumption occurs after approximately a microsecond, mainly through reactions with O_2_ and GSH, with smaller contributions from ascorbate (AH^−^) and α-tocopherol (TOH). The two dotted lines depict the contributions of reactions (R14) (R^●^ + GSH → GS^●^ + RH) and (R38) (R^●^ + TOH → TO^●^ + RH), assuming the absence of R^●^ from the ‘direct’ ionization of RH. As observed, the initial bio-radical R^●^ concentration of 2.5 μM, as utilized in this study, only becomes significant at times exceeding ~10 ms; panel (**b**) indicates that ROO^●^ is primarily formed on the microsecond timescale via reaction (2) (R^●^ + O_2_ → ROO^●^). Its consumption, occurring after tens of microseconds, is largely driven by reactions with ascorbate and nitric oxide (^●^NO), with reactions involving GSH and TOH playing a minor role. For reference, the dashed line at ~0.2 μs marks the transition from nonhomogeneous spur kinetics to homogeneous kinetics in the absence of dose rate effects.

To clarify the molecular mechanisms underlying radiolysis in our modeled bulk cell water described above, Figure 1 presents the time profiles of the yields of the key chemical species involved in the process. As can be seen, carbon-centered free radicals (R^●^) and peroxyl radicals (ROO^●^) are primarily produced, with R^●^ forming via reaction (1) and ROO^●^ via reaction (2) during oxygen consumption. Conversely, cellular biomolecules (RH) and oxygen are primarily consumed (“depleted”) through reactions (1) and (R1) and reaction (2), respectively. These findings underscore the dominant role of R^●^ in O_2_ consumption within the 1–100 μs range. Figure 2a,b reinforces this by displaying the time-dependent extents Δ*G*(R^●^) and Δ*G*(ROO^●^), expressed as molecules per 100 eV, for each reaction contributing to *G*(R^●^) and *G*(ROO^●^) from 1 ps to 1 s, based on our Monte Carlo simulations.

Figure 2a reveals that glutathione (GSH) and ascorbate (AH^−^) also contribute to R^●^ removal in this time range, though less efficiently. Figure 2b illustrates the role of cellular antioxidants in scavenging ROO^●^ radicals, with ascorbate and nitric oxide (^●^NO) being the most effective, while GSH and α-tocopherol (TOH) play minor roles. Efficient scavenging between 10 μs and ~1 ms underpins the bell-shaped *G*(ROO^●^) curve, peaking at ~50 μs and disappearing after 1 ms (see Figure 1). This rapid antioxidant response effectively minimizes ROO^●^-mediated lipid peroxidation, oxidative stress, and DNA damage beyond the millisecond scale. Ascorbate, ^●^NO, GSH, and TOH act as potent radioprotectors by rapidly neutralizing radiation-induced peroxyl radicals, thereby significantly mitigating oxidative damage. These results challenge the findings of Labarbe et al. [47,62], who attributed FLASH radioprotection mainly to radiolytic ROO^●^ neutralization through R^●^ and ROO^●^ radical–radical combination or recombination. Our data indicate that these reactions only have a limited impact due to slower kinetics. Baikalov et al. [88] reached similar conclusions. This, in turn, underscores the critical role of fast-reacting cellular antioxidants, especially ascorbate and nitric oxide, in achieving effective FLASH radioprotection. These antioxidants not only neutralize radiation-induced peroxyl radicals quickly but also prevent downstream oxidative damage, thereby maintaining cellular integrity during FLASH irradiation.

**Figure 3:**
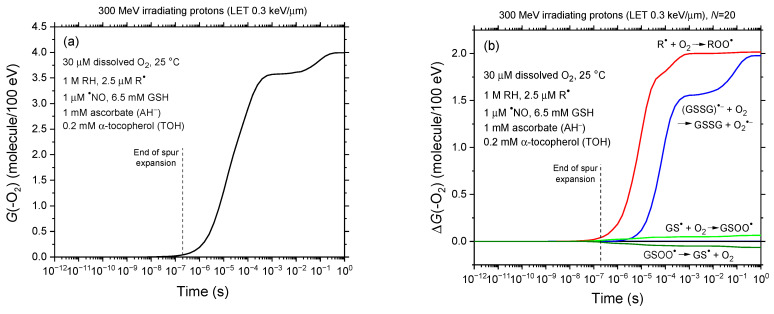
Panel (**a**): Time evolution of the oxygen consumption yield, *G*(–O_2_), obtained from our IONLYS-IRT Monte Carlo track chemistry simulations of radiolysis in our modeled cell-like water containing 30 μM dissolved oxygen at 25 °C. The simulations covered timescales from 1 ps to 1 s, employing 300-MeV incident protons (LET ~ 0.3 keV/μm) at various dose rates, each pulse involving 20 to 50 irradiating protons. The resulting *G*(–O_2_)-vs.-time curves remain nearly superimposed independent of the value of *N* (see text). Panel (**b**): Time-dependent extents Δ*G*(–O_2_), expressed in molecules per 100 eV, for the individual reactions contributing to *G*(–O_2_) from 1 ps to 1 s, based on our Monte Carlo simulations. Oxygen consumption primarily occurs through reactions with R^●^ and GSSG^●−^ radicals. The irradiation conditions are identical to those described in the caption of Figure 1. For reference, the dashed line at ~0.2 μs indicates the transition from nonhomogeneous spur kinetics to homogeneous kinetics in the absence of dose rate effects.

Figure 3a presents the time evolution of the oxygen consumption yield, *G*(–O_2_), during the radiolysis of our modeled cell-like water containing 30 μM dissolved oxygen at 25 °C, over a timescale from 1 ps to 1 s. Figure 3b provides a detailed breakdown of the time-dependent contributions, Δ*G*(–O_2_), of individual reactions to *G*(–O_2_), based on our Monte Carlo simulations. As expected, *G*(–O_2_) remains at zero during the first moments since O_2_ is not a primary species in low-LET water radiolysis. However, it begins to increase once dissolved O_2_ reacts with R^●^ and GSSG^●−^ (glutathione disulfide radical anion) via reactions (2) and (R23). In this process, *G*(–O_2_) rises rapidly, reaching a plateau of about 4*G* units around 1 s. Notably, the involvement of reaction (R23) (GSSG^●−^ + O_2_ → GSSG + O_2_^●−^) in oxygen depletion has not been previously recognized; its contribution, nearly 2*G* units at ~1 s, is comparable to that of reaction (2). In contrast, reaction (R18) (GS^●^ + O_2_ → GSOO^●^) has a negligible impact.

Another noteworthy observation is that the *G*(–O_2_)-vs.-time curve in Figure 3a remains nearly unchanged regardless of the *N* values used in the simulations. This consistency arises because these *N* values (20 to 50) are sufficiently low to prevent track overlap before ~3 × 10^−8^–10^−7^ s (see Figure 6 of Alanazi et al. [69]). By this time, most R^●^ radicals have already formed via reaction (1) (RH + ^●^OH → R^●^ + H_2_O), as the reciprocal of the scavenging power of O_2_ at 30 μM towards these radicals ensures that reaction (1) occurs within 2 × 10^−9^ s. Similarly, the contribution of reaction (R23) remains largely unaffected by dose rate, since most glutathione thiyl radicals are generated through reaction (R17) with ^●^OH radicals before 10^−8^ s, well before dose rate effects become relevant. Through reaction (R22), these GS^●^ radicals subsequently react with GSH to form GSSG^●−^, which then reacts with O_2_ independently of any dose rate influence.

Using our calculated *G*(–O_2_) values from Figure 3a, we can then estimate the corresponding concentration of consumed (depleted) oxygen, [–O_2_], over time. This estimation follows the general relationship *C* = *ρDG*, where *C* is concentration, *ρ* is solution density, *D* is radiation dose, and *G* is chemical yield [147]. Assuming a uniform distribution of O_2_ molecules within the considered circular cylinder of length 1 μm and radius *R*_o_ = 0.1 μm (see Section 2.1), this concentration, expressed in millimolar, can be directly determined from the following equation [59,148,149,150]:

[–O_2_] ≈ 5.3 × 10^−4^ × *N* × LET × *G*(–O_2_), (8)

where LET ~ 0.3 keV/μm for 300-MeV irradiating protons, *N* is the number of protons per pulse, and *G*(–O_2_) is in molecules per 100 eV.

**Figure 4:**
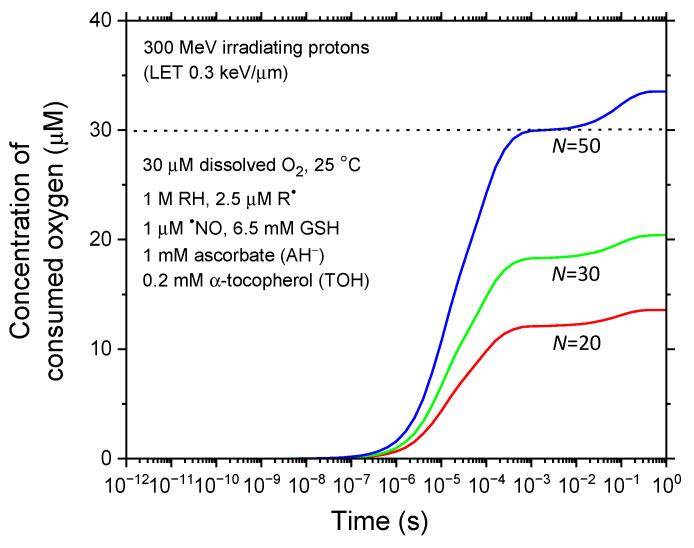
Time dependence of consumed (depleted) oxygen concentration ([–O_2_] in μM) for three values of *N*, the number of irradiated protons per pulse. The dotted line at 30 μM marks the chosen intracellular O_2_ concentration. The critical dose rate threshold for effective oxygen depletion and transient hypoxia, optimizing protective effects, is reached at *N* ≈ 50.

Figure 4 illustrates the time evolution of the corresponding consumed oxygen concentrations, calculated using Equation (8) for *N* = 20, 30, and 50, based on the *G*(–O_2_) values from our simulations (Figure 3a). As shown, [–O_2_] rises sharply from the microsecond range, reaching an initial, relatively prolonged plateau around 1 millisecond, followed by a second, slightly higher plateau near 1 s. These plateaus increase significantly, roughly proportional to the dose rate.

Notably, our model identifies a critical dose rate threshold below which the FLASH effect, as predicted by the ROD hypothesis, cannot fully manifest. As indicated in Figure 4, this threshold—corresponding to complete oxygen consumption—is reached at *N* = 50. In contrast, at *N* = 20, which represents typical FLASH irradiation conditions with 300 MeV incident protons (LET ~ 0.3 keV/μm) delivering ~30 Gy at an instantaneous dose rate of ~10^6^–10^7^ Gy/s, only a fraction (~13.6 μM or ~45.3%) of the intracellular oxygen (30 μM under our irradiation conditions) is consumed. This comparison with existing experimental data indicates that oxygen depletion alone—and thus the ROD hypothesis—is insufficient to fully explain the observed FLASH effect. These findings align with recent experimental reports challenging the hypothesis that ROD-induced radioresistance alone accounts for the FLASH tissue-sparing effect at clinically relevant doses and dose rates [52,53,54,55]. Nonetheless, our model suggests that transient oxygen depletion may play a partial role in the FLASH effect, underscoring the need to explore additional contributing factors.

**Figure 5:**
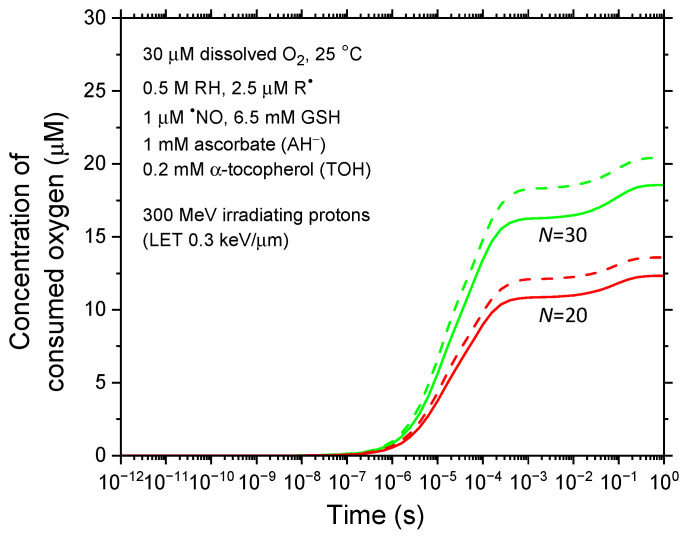
Time evolution of consumed (depleted) oxygen concentration ([–O_2_] in μM) for two values of *N*, the number of irradiated protons per pulse, using [RH] = 0.5 M (solid lines). For comparison, [–O_2_] values obtained with [RH] = 1 M (see Figure 4) are depicted as dashed lines. Both simulation scenarios assume an intracellular O_2_ concentration of 30 μM.

**Figure 6:**
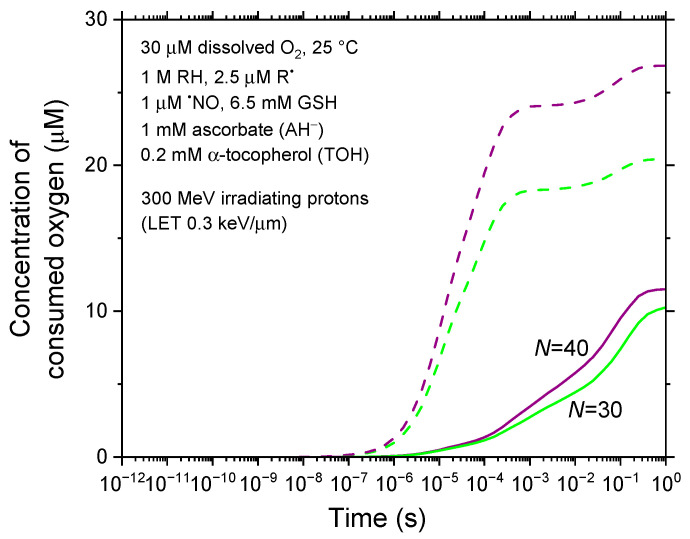
Time evolution of consumed (depleted) oxygen concentration ([–O_2_] in μM) for two values of *N*, the number of irradiated protons per pulse, calculated in a diffusion environment of bulk cell water where all reactive species have diffusion coefficients reduced by a factor of 100 relative to pure liquid water (solid lines). For comparison, [–O_2_] values obtained using molecular diffusion in pure liquid water (see Figure 4) are shown as dashed lines. Both simulation scenarios assume an intracellular O_2_ concentration of 30 μM.

Figure 5 and Figure 6 examine the sensitivity of our results to two key parameters: the concentration of carbon-based biomolecules (RH), accounting for some cellular heterogeneity, and the diffusion coefficients of reactive species, acknowledging that molecular diffusion in bulk cell water differs significantly from that in pure liquid water. These analyses offer insight into how these factors influence the radiolytic dynamics and the extent of radiolytic oxygen depletion, further refining our understanding of the underlying mechanisms.

In Figure 5, we evaluate the impact of reducing the intracellular bio-organic molecule concentration [RH] from 1 M to 0.5 M on oxygen consumption under representative FLASH irradiation conditions. Using 300 MeV incident protons (LET ~ 0.3 keV/μm) with *N* = 20 and 30 irradiating protons per pulse, we observe that the depleted oxygen concentration [–O_2_] is lower when [RH] is reduced to 0.5 M compared to 1 M. Specifically, for *N* = 20, which corresponds to delivering ~30 Gy at an instantaneous dose rate of ~10^6^–10^7^ Gy/s, [–O_2_] decreases from ~13.6 μM to 12.3 μM when [RH] is lowered from 1 M to 0.5 M—representing a ~10% decrease. This reduction in oxygen consumption is expected, as a lower [RH] decreases the concentration of R^●^ radicals through reaction (1) RH + ^●^OH → R^●^ + H_2_O, thereby reducing the formation of peroxyl radicals (ROO^●^) via reaction (2) R^●^ + O_2_ → ROO^●^ (see Figure 3b).

All simulations presented thus far have assumed that the diffusion coefficients of all reactive species correspond to their values in pure liquid water at room temperature. However, it is well established that intracellular proton (H^+^) mobility is approximately 100–300 times slower than in free water [151,152,153]. To account for the diffusion environment of bulk cell water, we explore a scenario in which the diffusion coefficients of all reactive species are reduced by a factor of 100 relative to their values in pure liquid water. Figure 6 compares the time-dependent consumed O_2_ concentrations for *N* = 30 and 40 irradiated protons per pulse under these modified diffusion conditions with those from Figure 4, using identical parameters. Notably, we observe a significant reduction in [–O_2_], which decreases from 20.4 μM to 10.3 μM for *N* = 30 at 1 s—representing a more than 50% decrease. This reduction in [–O_2_] is even more pronounced for *N* = 40.

These findings, considering both cellular heterogeneity and molecular diffusion in bulk cell water, consistently indicate that the ROD hypothesis alone cannot fully explain the FLASH effect.

## 4. Conclusions

A leading explanation for the FLASH effect in radiotherapy, the ROD hypothesis, posits that an ultra-short radiation pulse transiently “depletes” intracellular oxygen through radiolytic consumption. This leads to acute hypoxia, temporarily enhancing cellular resistance to radiation. Despite widespread acceptance, the ROD hypothesis continues to be debated. Specifically, the debate primarily focuses on whether the amount of O_2_ depleted during pulse irradiation is substantial enough to cause significant hypoxia in well-oxygenated tissues in vivo, and whether this can fully account for the observed protective effects of FLASH-RT.

In this study, we employed a computational model to evaluate the ROD hypothesis from a radiation–chemical perspective. We utilized our multi-track chemistry Monte Carlo simulation code, IONLYS-IRT, optimized to model the effects of dose rate on radiolysis within a homogeneous, aerated aqueous environment, mimicking a confined cellular space under instantaneous proton pulses. This medium primarily consisted of water, carbon-based biological molecules (RH), radiation-induced bio-radicals (R^●^), glutathione (GSH), ascorbate (AH^−^), nitric oxide (^●^NO), and α-tocopherol (TOH). Our model precisely tracked temporal changes in these components, with a special focus on O_2_ consumption, from the initial picoseconds up to one second post-exposure.

Among our key findings, the simulations revealed that cellular oxygen is transiently depleted primarily through reactions with R^●^ radicals and, to a similar extent, with glutathione disulfide radical anions (GSSG^●−^). Contrary to previous reports, we found that the resulting peroxyl radicals (ROO^●^) are not neutralized via recombination reactions but are instead rapidly scavenged by antioxidants in irradiated cells, notably AH^−^ and ^●^NO. These antioxidants effectively prevent the propagation of damaging peroxidation chain reactions. Thus, our results suggest that antioxidants play a critical role in the FLASH effect, mitigating radiation-induced cellular damage and enhancing radioprotection.

Most importantly, our model identified a critical dose rate threshold below which the FLASH effect, as predicted by the ROD hypothesis, cannot fully manifest. By comparing our results with existing experimental data, we find that the ROD hypothesis alone does not entirely account for the FLASH effect observed in radiotherapy. Our analysis, which also incorporates cellular heterogeneity and molecular diffusion in bulk cell water, consistently suggests that the ROD hypothesis is insufficient to fully explain the FLASH effect. This underscores the complex interplay of cellular responses to high dose-rate radiation and indicates that additional mechanisms or factors, such as alternative oxygen depletion pathways or dynamic cellular responses, are likely involved.

Further research is warranted to identify and quantify these factors that could complement the ROD hypothesis, aiming to provide a more comprehensive understanding of the conditions required to trigger the FLASH effect.

## Data Availability

Data generated or analyzed during this study are provided in full within the article.
